# Binocular Advantage in Established Eye–Hand Coordination Tests in Young and Healthy Adults

**DOI:** 10.3390/jemr18030014

**Published:** 2025-05-07

**Authors:** Michael Mendes Wefelnberg, Felix Bargstedt, Marcel Lippert, Freerk T. Baumann

**Affiliations:** Department 1 of Internal Medicine, Center for Integrated Oncology Aachen-Bonn-Cologne-Düsseldorf, University Hospital Cologne, 50379 Cologne, Germany; felixbargstedt@yahoo.de (F.B.); marli-94@web.de (M.L.); freerk.baumann@uk-koeln.de (F.T.B.)

**Keywords:** eye–hand coordination tests, validity, monocular, stereopsis, assessment

## Abstract

Background: Eye–hand coordination (EHC) plays a critical role in daily activities and is affected by monocular vision impairment. This study evaluates existing EHC tests to detect performance decline under monocular conditions, supports the assessment and monitoring of vision rehabilitation, and quantifies the binocular advantage of each test. Methods: A total of 70 healthy sports students (aged 19–30 years) participated in four EHC tests: the Purdue Pegboard Test (PPT), Finger–Nose Test (FNT), Alternate Hand Wall Toss Test (AHWTT), and Loop-Wire Test (LWT). Each participant completed the tests under both binocular and monocular conditions in a randomized order, with assessments conducted by two independent raters. Performance differences, binocular advantage, effect sizes, and interrater reliability were analyzed. Results: Data from 66 participants were included in the final analysis. Significant performance differences between binocular and monocular conditions were observed for the LWT (*p* < 0.001), AHWTT (*p* < 0.001), and PPT (*p* < 0.05), with a clear binocular advantage and large effect sizes (SMD range: 0.583–1.660) for the AHWTT and LWT. Female participants performed better in fine motor tasks, while males demonstrated superior performance in gross motor tasks. Binocular performance averages aligned with published reference values. Conclusions: The findings support the inclusion of the LWT and AHWTT in clinical protocols to assess and assist individuals with monocular vision impairment, particularly following sudden uniocular vision loss. Future research should extend these findings to different age groups and clinically relevant populations.

## 1. Introduction

Conditions such as macular degeneration, strabismus, amblyopia, diabetic retinopathy, or malignant eye diseases can lead to diminished or complete loss of binocular vision. Between 7 and 9 percent of the US population are stereo blind [1]. This condition can lead to impaired activities of daily living and ultimately social isolation tendencies and symptoms of anxiety and depression [2,3,4,5]. These consequences mainly result from absent or diminished stereopsis, a mechanism responsible for depth perception derived from the disparities in visual information between the two eyes. Stereopsis enables the discernment of minute variations in depth at close distances and facilitates swift and accurate reactions, particularly important in tasks requiring rapid motor responses [6].

The functional significance of stereopsis in everyday activities has been extensively studied in previous investigations [7,8,9,10,11,12]. Notably, compromised or absent stereopsis is linked to diminished eye–hand coordination (EHC) [10,11]. EHC denotes the brain’s ability to interpret visual input from the eyes and efficiently guide hand movements [13]. In previous investigations, absent or deficient stereopsis was linked to impaired EHC during activities such as object grasping, placement, and pouring drinks [8,11,12]. Ninety percent of patients affected report a lack of specific assessments, supportive care, or formal guidance on coping strategies for this condition in their daily lives [2,14]. In two recent case studies, we have demonstrated that stereopsis-related decline in EHC can be compensated through targeted rehabilitation focusing on improving general EHC performance [15,16].

Nonetheless, there is currently no established protocol for a clinically relevant EHC assessment linked to uniocular vision impairment nor reliable norms for relevant EHC tests. In order to effectively identify individuals requiring targeted rehabilitation and to monitor their progress, there is a pressing need for EHC tests sensitive to the stereoscopic aspect of EHC. Precise EHC tests applied in experimental studies involving eye-tracking and video analysis are not feasible in clinical settings constrained by personal resources and time, and these tests do not allow for the classification of severity [8,9,10]. Moreover, most previous investigations involve stereo-deficient but not stereo-blind individuals, individuals with limited coordinative abilities, or children, hence limiting generalizability of findings [10,11]. In order to reliably detect performance discrepancies in stereo blindness in adults, the primary objective of this study was to evaluate existing EHC tests in healthy young adults with no coordinative limitations under mono- and binocular condition.

## 2. Materials and Methods

### 2.1. Participants

To minimize potential distortion of results from eye diseases or neuromuscular coordination issues, we selected a sample of young healthy individuals with relative consistency in overall coordination skills. Participants were sports and exercise science students recruited at the German Sports University in Cologne between February and June 2023. They were recruited conveniently on campus via direct contact [17]. The eligibility criteria encompassed student of sports and exercise science up to 30 years of age with good physical and mental health conditions (e.g., absence of orthopedic, neurological, psychological, and neuromuscular disorders) and overall intact stereopsis as well as the absence of ophthalmological conditions, especially strabismus and amblyopia. To assess for stereopsis, the Lang Stereo-Test I (LST) was utilized. The test shows three test images (cat, star, and car) on a postcard-sized surface, which are presented at different disparities of 1200, 600, and 550 arc seconds, at a distance of approx. 40 cm [18]. Recognition of 2/3 images was considered as overall intact stereopsis capacity. Subjects that did not meet the eligibility criteria were excluded from participation. To ensure high and lasting motivation, participants were offered prices of EUR 50, 20, and 10 for the first, second, and third place, respectively, in the overall performance of the tests applied. All subjects included gave informed consent for study participation and publication of results. On the test occasion, participants initially underwent a brief anamnesis including age, sex, vision defects, eye dominance (ring bearing test), hand dominance, and ball sports engagement. The ring bearing test is a practical test that involves looking at the examiner’s nose in a distance of three meters through a circle of roughly five cm diameter, created by the subject via overlapping hands with arms outstretched. The eye that is visible through the circle is the dominant eye [19].

An a priori sample size calculation for paired *t*-tests with an estimated medium effect size of 0.5, an alpha error of 0.05, and power of 0.80 using the G*Power tool [20] yielded a minimum sample size of 34.

This investigation was part of the EyeCanMoveiT project (Clinical Trial Register ID: DRKS00031207). The study was approved by the Ethics Committee of the University Hospital of Cologne (No. 22-1062_1) and conducted in accordance with institutional regulations and the standards of the Declaration of Helsinki.

### 2.2. Eye–Hand Coordination Assessment

All assessments were conducted at a prepared facility on campus. We involved two raters, including one physiotherapist one exercise science professional, to evaluate the applicability of our modified test protocols, which are described below for each EHC test, separately, for uniocular performance assessment. Each test was administered under four different conditions (uniocular, rater 1; binocular rater 1; uniocular, rater 2; binocular, rater 2) and twice per condition. The average for each condition was utilized for statistical evaluation. Assessment for the non-dominant eye was evaded as a previous investigation showed small or no performance differences for these conditions [12]. To avoid systematic training or fatigue effects, the conduction sequence for the EHC tests was randomized for each rater using a list-based simple randomization technique. Between each rater’s assessment, a 30 min break was given.

**Figure 1:**
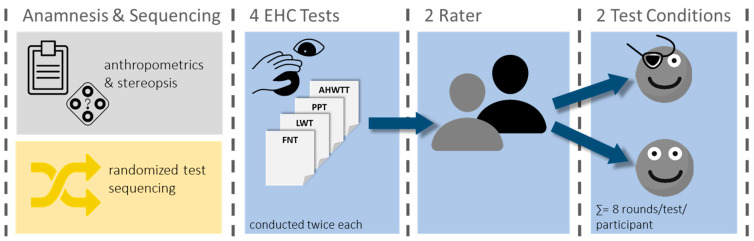
Assessment procedure. Abbreviations: EHC, eye–hand coordination; AHWTT, Alternate Hand Wall Toss Test; PPT, Purdue Pegboard Test; LWT, Loop-Wire Test; FNT, Finger–Nose Test.

The EHC tests were selected based on a comprehensive scoping literature review (in preparation for submission). All chosen tests were required to potentially involve three-dimensional perception at close range and be easily conducted within time-constrained settings. Additionally, the criteria for selection included fine and gross EHC coordination and/or tasks involving the use of tools for solving. The four tests meeting these criteria were the Purdue Pegboard Test (PPT), the Finger–Nose Test (FNT), the Alternate Hand Wall Toss Test (AHWTT), and the Loop-Wire Test (LWT). The entire assessment procedure is outlined in Figure 1.

The PPT, originally designed in 1949 by Lafayette Instruments, Lafayette, IN, USA, is a well-established manual dexterity test [21]. It has been validated for various applications, with normative values for healthy adults aged above 40 [22] and adolescent aged between 14 and19 [23] as well as for seniors with low vision [24]. This test requires the subject, while sitting in an upright position at a table, to insert as many pins as possible into a vertical column of holes on a board within a 30-second period, in our set up, using their dominant hand only. The rating protocol, in contrast to the manufacture’s instruction comprised only of two rounds per condition to calculate average performance.

The FNT is a widely accepted clinical measure of upper limb performance, although it has yet to be validated for use in individuals with visual impairments. The variety of test procedures is described in detail elsewhere [25]. In our set up, subjects have to sit in an upright position on a chair, with a table tennis ball placed at shoulder level on the dominant hand’s side, at an arm’s length away from the subject. Then, the nose and ball have to be touched with the fingertip of the index finger of the dominant hand in alternation as many times a possible in 30 s.

The AHWTT is a conventional assessment tool utilized in sports medicine for evaluating eye–hand coordination [26]. During the test, participants throw a ball from one hand in an underarm motion toward a wall positioned 2 m away. They then endeavor to catch the ball with the opposite hand as frequently as possible, alternating between hands, all within a 30-second timeframe. In our set-up, a bowl with 9 balls was placed next to the participants at hip level on their dominant hand’s side. They were instructed to pick a new ball in case they dropped one. Furthermore, only the upper extremities were permitted to be used for catching. Any other involvement of the upper body was not permitted (e.g., pinching the ball between the hand and upper body).

The LWT, also known as the hot or buzz wire test, is a relatively novel EHC test that has demonstrated strong discriminatory ability between binocular and monocular performance in previous publications [7,12]. In our setup, it involves moving a stick with an attached wire loop along a three-dimensional oriented wire track without making contact. When the wire is touched, a beeping noise occurs, and subjects have to remove the loop from the wire before continuing. The number of contacts and the time taken to complete the task are recorded. Noteworthy, the first seven participants had to be excluded from calculation for the LWT performance due to necessary alterations to the test protocol. Initially, participants had to restart from the beginning whenever they touched the wire, which led to rampant test completion times. This was then modified to a time and contact evaluation.

### 2.3. Statistics

Statistical analysis of the findings was conducted using IBM SPSS Statistics for Windows, Version 27.0 (IBM Corp., New York, NY, USA). To assess performance differences between binocular and uniocular conditions, either paired *t*-tests or non-parametric Wilcoxon tests were employed following Shapiro–Wilk normal distribution testing. Additionally, binocular advantage for each test including effect size were calculated based on rater 1 assessments.

Sex-dependent differences in performance were evaluated via unpaired *t*-test or Mann–Whitney U-test depending on distribution of EHC test results. Additionally, potential moderating effects of ball sports engagement were analyzed using regression analysis.

Interrater reliability analysis was performed using either Spearman rank correlation or Pearson product-moment correlation, depending on the normality of the distribution of data. Extreme outliers above two standard deviations from the sample mean will be removed from any statistical calculations.

## 3. Results

Between February and June 2023, a total of 72 subjects were screened for eligibility. Two subjects were excluded due to lack of stereopsis capacity identified based on LST results. Seventy subjects underwent the complete testing procedure. Due to some extreme outliers, participants exhibiting binocular advantage values above two standard deviations from the sample mean were removed from all statistical analysis.

Therefore, the resulting sample assessed by rater 1 comprised 66 subjects. Forty-five subjects were assessed by both raters. There were no missing data. For the overall sample, in brief, the mean age was 22.36 (SD = 2.84), 42% were female, 97% had full stereopsis according to LST, and 47% reported ball sports engagement. All participants’ characteristics are listed in Table 1.

**Figure 2:**
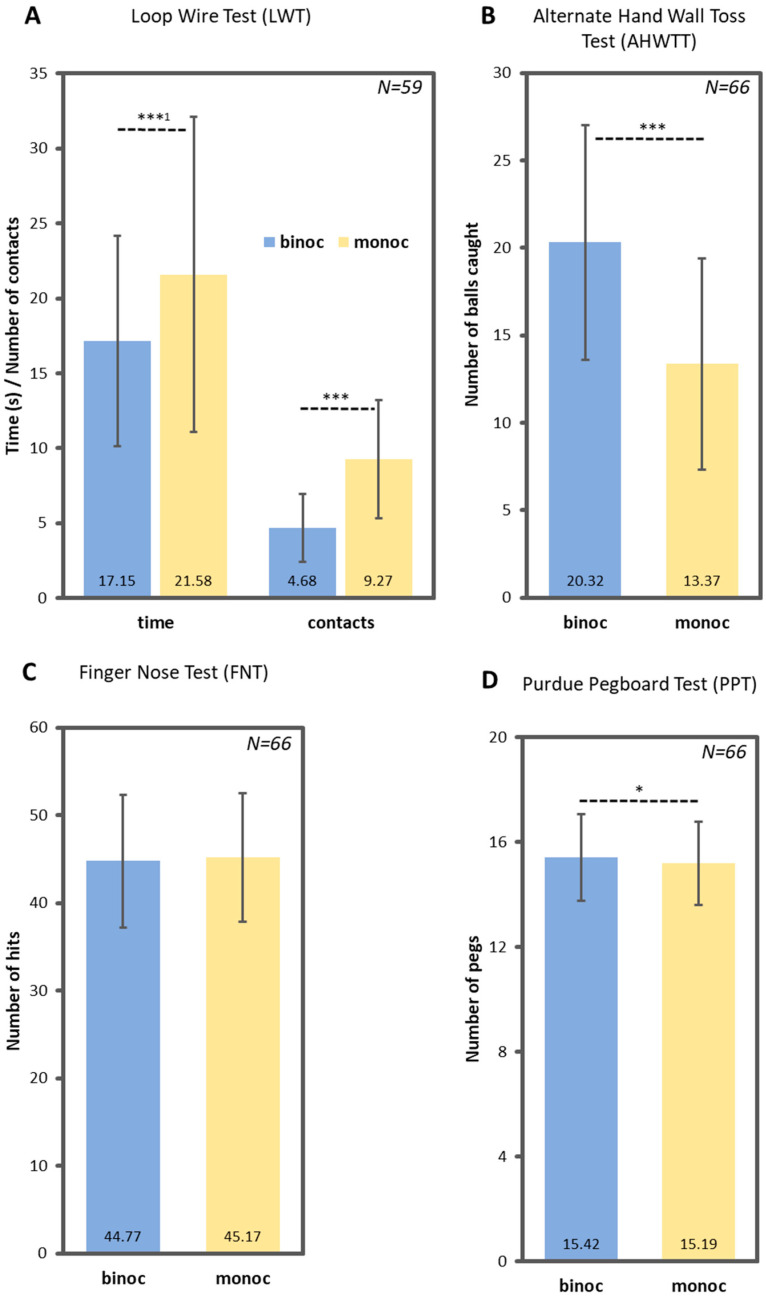
Mean and standard deviation for performance in EHC tests (**A**–**D**) with binocular versus monocular (dominant eye) vision. ***^1^ *p* < 0.001 (two-sided), calculated with Wilcoxon test. *** *p* < 0.001 (two-sided), calculated with paired *t*-test. * *p* < 0.05 (one-sided), calculated with paired *t*-test.

Regarding EHC performance under binocular versus monocular viewing, results demonstrate a large and highly significant (two-sided) difference for both LWT variables, LWTcontacts; t(58) = −10.268; *p* < 0.001; LWTtime; z = 6.628; *p* < 0.001, and AHWTT; t(65) = 13.488; *p* < 0.001. For the PPT, a significant (one-sided) difference was found, t(65) = 1.740; *p* = 0.043, whereas there were only tiny and non-significant differences for FNT; t(65) = −0.998; *p* = 0.161. Means and standard deviations are illustrated in Figure 2. There were no moderating effects regarding ball sports engagement.

Table 2 presents test results for female and male participants under binocular and monocular conditions. Females on average perform better in the PPT under both conditions, while males outperform females in the AHWTT. Notably, females show a binocular advantage in AHWTT, PPT, and FNT compared to males.

Interrater reliability was calculated based on smaller samples of 38–45 subjects depending on the test. The calculation yielded strong and highly significant correlations for all tests according to Pearson and Spearman (r > 0.6; *p* < 0.001), while AHWTT depicted strongest correlations for both conditions, namely, monocular and binocular. See Table 3 for details.

**Figure 3:**
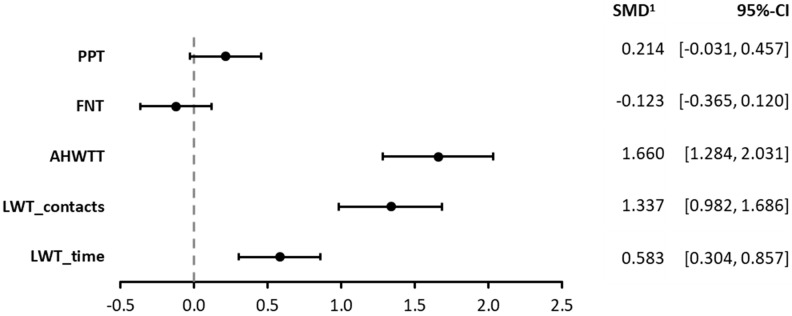
Forest plot of effect sizes for binocular versus monocular performance differences in EHC tests. Values above zero indicate a binocular advantage, and values below zero indicate a binocular disadvantage accordingly. Values for LWT have been multiplied by −1 in order to standardize for binocular advantage. Abbreviations: SMD, standard mean difference; LWT, Loop-Wire Test; AHWTT, Alternate Hand Wall Toss Test; PPT, Purdue Pegboard Test; FNT, Finger–Nose Test. 1 The standard mean difference (SMD) according to Cohen’s d.

Concerning a binocular advantage, which is defined as the ratio of binocular versus monocular performance, LWT_contacts_ demonstrated the highest mean value, followed by AHWTT, LWT_time_, and, with only minor average advantage, the PPT. A slightly average disadvantage was observed for FNT. Notably, LWT and AHWT exhibited high dispersion with standard deviations ranging from 30.1% (LWT_time_) to 54.6% (LWT_contacts_) of the mean value, while standard deviations for PPT and FNT were around 0.07% of the mean value. Interestingly, effect size calculations according to Cohen’s d yielded, in descending order, high effect sizes for AHWTT (1.660) and LWT_contacts_ (1.337), a medium effect size for LWT_time_ (0.583), a small effect size for PPT (0.214), and a small and negative effect size for FNT (−0.123). However, the only significant effects (CI below or above 0) were found for LWT and AHWTT. Notably, effect sizes for LWT were multiplied by −1 to simplify comparison between values depicting binocular advantage and those depicting disadvantage. Results of binocular advantage calculations are illustrated in Figure 3.

Also, we conducted correlation analysis for all EHC tests under both conditions, monocular and binocular, to check for similarities in EHC capacities measured by the assessment instruments. Overall, all correlations, significant and non-significant, except for one case, were very low to low (0.03–0.29), implying that the conducted tests measure disparate aspects of EHC. There were only a few significant results. AHWTT in binocular condition exhibited a significant (*p* < 0.05) negative correlation of r = −0.29 with PPT (calculated using Pearson product-moment correlation). Under monocular conditions, AHWTT demonstrated a very significant (*p* = 0.006) positive correlation of r = 0.33 with the FNT. The only strong correlation found was between LWT_time_ and LWT_contacts_ under monocular conditions (r = 0.667; *p* < 0.001). Table 4 lists all correlation values for the EHC tests.

## 4. Discussion

The results of this investigation provide valuable insights into the potential clinical relevance of various EHC tests in identifying performance declines associated with diminished binocular vision. To our knowledge, this is the largest study on uniocular EHC across multiple tests. We demonstrated that two of the tests conducted, the Loop-Wire Test (LWT) and the Alternate Hand Wall Toss Test (AHWTT), are sensitive to performance declines under uniocular versus binocular conditions. The binocular advantage for these tests span from 26 to 129 percent. The AHWTT reached the highest effect sizes with lower dispersion of results in comparison to the LWT. Next, the inter-rater reliability analysis indicates robustness of the tests in the present context with strong and highly significant (*p* < 0.001) correlation coefficients in all four tests.

Unlike previous investigations [10,11,12], we tested a large and consistent sample of young and healthy subjects (students of sports and exercise science) with above average motor performance skills. Thereby, we overcame potential bias resulting from large heterogeneity in manual dexterity or neurologic, psychological, orthopedic, and ophthalmologic conditions. The evaluated tests encompass a broad range of EHC demands, including grasping and placing objects, throwing and catching, targeting motions, and tool use. Our findings suggest that binocular vision plays a varying role in these tasks, as indicated by the generally low and non-significant correlations between test results under both conditions.

The AHWTT revealed a significant binocular advantage in ball-catching performance. Stereopsis plays a crucial role in estimating a ball’s trajectory, speed, and distance [27]. Several studies support this finding, demonstrating superior performance in binocular compared to monocular ball-catching tasks [28,29]. Interestingly, male participants performed better than female participants. This was also reflected in the binocular advantage of the task, exhibiting a 50% advantage for male participants and nearly 100% for female participants. Previous research supports this observation by demonstrating higher performance in tasks requiring aiming accuracy in men over women [30]. Ultimately, Mazyn et al. [31] observed that monocular ball-catching performance can per se improve through intensive training, as shown in a small sample of stereo-blind individuals (N = 6) compared to a control group (N = 8). This supports the idea that improved ball catching in monocularity indicates compensatory mechanism development, highlighting the AHWTT as a potential clinical measure of EHC in uniocular conditions.

The LWT results, consistent with previous studies [7,12], confirm a clear binocular advantage, with female participants generally performing better than males. The test likely benefits from motion-in-depth disparity processing and vergence control, where stereopsis aids depth perception along the wire track, and vergence primarily stabilizes fixation and reduces response latency. LWT performance relies on specific EHC aspects that monocular cues cannot fully compensate for (e.g., shading, perspective, size, interposition, motion parallax). The high dispersion of LWT contacts observed in some subjects may be due to upper limb trembling, which also affected all excluded participants, suggesting tremor as a limiting factor. Devi et al. [7] found no improvement in LWT performance with habituation or training, as sudden monocular occlusion and permanent uniocularity produced similar results, reinforcing the superiority of binocular performance. However, broad age variation (6–37 years) and sex distribution differences (22:7 in the uniocular vs. 11:19 in the binocular group) complicate comparisons. Our findings align with prior research [22,24,30], showing women generally excel in fine motor control tasks. Additionally, eye patches were not applied based on dominance, a factor found to influence results [12], limiting the generalizability of performance equality between groups. Overall, the LWT may not be suitable for monitoring vision rehabilitation in cases of complete uniocular vision loss due to its limitations in assessing individuals with upper limb instability and the absence of valid monocular coping mechanisms. However, in cases of reduced stereopsis, it may still be useful for evaluating optometric interventions and binocular vision integration into EHC tasks.

The PPT, despite its widespread use in assessing manual dexterity, showed only one-sided significance in performance differences and a small, non-significant effect size, indicating limited sensitivity to subtle binocular advantages in EHC. To our knowledge, this is the largest study on the PPT for young healthy adults both under binocular and monocular conditions. Significant sex differences of approximately two pegs in both binocular and monocular conditions align with prior studies on healthy and visually impaired older adults [22,24]. While no normative values exist for individuals aged 20–30 years, our results match those for 40- to 49-year-olds reported by Agnew et al. [22] in binocular conditions for men, while women in our study outperformed this benchmark by 5% (15.9 vs. 16.7 pegs). Mathiowetz et al. [23] also found that females generally outperformed males in a study of 176 individuals aged 14–19 years, with performance improving with age. The only study demonstrating significant performance differences between stereo-intact and stereo-blind individuals in a pegging task [10] used head fixation and slanted peg holes, preventing compensatory head movements. Similarly, a recent small sample study (N = 9, mean age 24.3 ± 2.0 years) reported slightly higher dominant-hand performance (16.1 ± 1.9 pegs) than Agnew et al. [22] but found no significant correlation with depth perception [32], a result that should be interpreted cautiously given the small sample size. Nonetheless, given its high reliability and established norms [22,24,33,34], the PPT may still be valuable in clinical test batteries for general manual dexterity assessment. Two recent case studies on one young and one middle aged patient with stereopsis loss due to choroidal melanoma suggest that the PPT might be capable of detecting EHC declines induced by deteriorated visual capacity as well as EHC improvements through targeted visuomotor training [15,16].

Results for the FNT corroborate its unreliability to assess EHC performance declines in uniocular vision possibly due to the nature of the test itself, which may not necessitate fine three-dimensional perception and only demands gross visual orientation.

The interpretation of our findings is constrained by the selective and convenient sampling method used. More precisely, students of sports and exercise science with above average coordination skills and overall physical fitness do not reflect the general age-related reference population. This might limit our results in terms of representativeness and generalizability. Furthermore, it remains unclear whether our results are generalizable to the broader clinical population affected by sudden uniocular vision loss or whether they may be confounded by the inclusion of older participants with multiple chronic conditions affecting vision and motor skills. Specifically, conditions such as restlessness, tremor, carpal tunnel syndrome, or any other condition affecting upper limb performance could limit the applicability of our assessments. For the LWT, we had to exclude participants in our original protocol that demonstrated restlessness in their upper limb. Additionally, the sensitivity of the EHC tests in detecting stereoscopic vision loss in individuals with otherwise intact binocular vision, such as those with uveal melanoma treated with radiation, remains uncertain. It is also unknown if similar findings would emerge in other populations. Nonetheless, we did not conduct intra-rater reliability analysis. Thus, the consistency of test results over time is unclear, and test reliability cannot be assumed definitely.

## 5. Conclusions

Our investigation corroborates the need for targeted EHC assessments that are sensitive to binocular vision deficits, advocating for the inclusion of tests like the LWT and AHWTT in clinical protocols to better identify and support individuals with uniocular vision impairment. In contrast, the PPT showed limited sensitivity to binocular advantages but remains useful for general manual dexterity assessment, while the FNT proved useless for detecting EHC deficits in uniocular conditions. Notably, significant sex-dependent differences were observed across multiple tests, with women generally outperforming men in fine motor tasks and men excelling in ball-catching performance, aligning with previous research. The observed low correlations between different EHC tests emphasize the need for a multifaceted approach in comprehensive EHC assessment. Despite the strengths of our study, including a large and homogeneous sample, its generalizability to clinical populations with sudden or progressive uniocular vision loss and motor deficits in the upper limb remains uncertain. Further research is needed to validate the applicability of these tests in diverse populations, including individuals with acquired monocular vision loss due to disease or injury. Additionally, intra-rater reliability analysis is required to establish the long-term consistency of these tests in clinical and rehabilitative settings.

## Figures and Tables

**Table 1:** Characteristics of study participants by cohorts. Abbreviations: M, mean; SD, standard deviation.

Sample	Rater 1	Rater 1 and 2
N	66	45
Age (years), M (SD)	22.4 *(2.8)*	21.7 *(2.4)*
Sex, N (%)		
*Female*	28 *(42)*	17 *(38)*
*Male*	38 *(58)*	28 *(62)*
Visual Impairment, N (%)		
*Myopia*	15 *(23)*	8 *(17)*
*Hyperopia*	6 *(9)*	5 *(12)*
*None*	45 *(68)*	32 *(71)*
Stereopsis (Lang Test), N (%)		
*Full*	64 *(97)*	43 *(96)*
*Limited* *(2/3 of images recognized)*	2 *(3)*	2 *(4)*
Eye Dominance, N (%)		
*Right*	42 *(64)*	27 *(60)*
*Left*	24 *(36)*	18 *(40)*
Ball Sports, N (%)		
*Yes*	31 *(47)*	22 *(49)*
*No*	35 *(53)*	23 *(51)*

**Table 2:** Eye–hand coordination test results by sex (dominant hand only). Abbreviations: M, mean; SD, standard deviation; LWT, Loop-Wire Test; AHWTT, Alternate Hand Wall Toss Test; PPT, Purdue Pegboard Test; FNT, Finger–Nose Test.

	Male (N = 38)		Female (N = 28)	
	M	SD	M	SD
Binocular				
LWT_time_^1^	17.28	7.86	16.78	5.87
LWT_contacts_^1^	4.49	2.18	4.9	2.47
AHWTT *(balls)*	23.55	5.73	15.71	5.06
PPT *(pegs)*	14.60	1.52	16.71	1.19
FNT *(contacts)*	44.57	8.68	45.41	5.72
Monocular				
LWT_time_^1^	22.34	10.99	19.66	8.99
LWT_contacts_^1^	9.38	4.11	8.68	3.47
AHWTT *(balls)*	16.28	5.40	9.11	4.32
PPT *(pegs)*	14.69	1.51	16.08	1.23
FNT *(contacts)*	45.72	8.39	44.86	5.88
Binocular advantage ^2^				
LWT_time_^1^	1.33	0.44	1.17	0.31
LWT_contacts_^1^	2.39	1.22	2.16	1.33
AHWTT *(balls)*	1.51	0.34	1.97	0.68
PPT *(pegs)*	1.00	0.07	1.04	0.06
FNT *(contacts)*	0.97	0.07	1.02	0.07

^1^ N = 34 for male, and N = 25 for female. ^2^ Values above 1 indicate a binocular advantage.

**Table 3:** Results for interrater reliability correlation analysis. Abbreviations: LWT, Loop-Wire Test; AHWTT, Alternate Hand Wall Toss Test; PPT, Purdue Pegboard Test; FNT, Finger–Nose Test; bin, binocular; mon, monocular.

LWT (N = 38)				AHWTT (N = 45)		FNT (N = 45)		PPT (N = 45)	
Bin		Mon		Bin	Mon	Bin	Mon	Bin	Mon
Time	Contacts	Time	Contacts						
0.83 ^1^ ***	0.73 ^2^ ***	0.68 ^1^ ***	0.66 ^2^ ***	0.88 ^2^ ***	0.82 ^2^ ***	0.60 ^2^ ***	0.65 ^2^ ***	0.69 ^2^ ***	0.67 ^2^ ***

^1^ Spearman rank correlation. ^2^ Pearson product-moment correlation. *** *p* < 0.001.

**Table 4:** Results for Person product-moment and Spearman rank correlation analysis under monocular and binocular conditions for all EHC tests. Abbreviations: LWT, Loop-Wire Test; AHWTT, Alternate Hand Wall Toss Test; PPT, Purdue Pegboard Test; FNT, Finger–Nose Test.

	Binocular					Monocular				
	(N = 66)			(N = 59)		(N = 66)			(N = 59)	
	**AHWTT**	**FNT**	**PPT**	**LWT**		**AHWTT**	**FNT**	**PPT**	**LWT**	
				**Time**	**Contacts**				**Time**	**Contacts**
LWT										
Time	−0.16 ^1^ (0.217)	−0.06 ^1^ (0.676)	−0.05 ^1^ (0.712)	1.00 ^1^	0.19 ^1^ (0.154)	−0.08 ^1^ (0.559)	−0.15 ^1^ (0.243)	−0.03 ^1^ (0.852)	1.00 ^1^	0.67 *** ^1^ (<0.001)
Contacts	0.03 (0.810)	−0.06 (0.670)	−0.08 (0.547)	0.19 ^1^ (0.154)	1.00	0.03 (0.836)	−0.13 (0.322)	−0.09 (0.491)	0.67 *** ^1^ (<0.001)	1.00
AHWTT	1.00	0.24 (0.055)	−0.29 * (0.017)	−0.16 ^1^ (0.217)	0.03 (0.810)	1.00	0.33 ** (0.006)	−0.22 (0.076)	−0.08 ^1^ (0.559)	0.03 (0.836)
FNT	0.24 (0.055)	1.00	0.24 (0.051)	−0.06 ^1^ 0.676	−0.06 0.670	0.33 ** (0.006)	1.00	0.23 (0.069)	−0.15 ^1^ 0.243	−0.13 (0.322)
PPT	−0.29 * (0.017)	0.24 (0.051)	1.00	−0.05 ^1^ (0.712)	−0.08 (0.547)	−0.22 (0.076)	0.23 (0.069)	1.00	−0.03 ^1^ (0.852)	−0.09 (0.491)

* *p* < 0.05. ** *p* < 0.01. *** *p* < 0.001. ^1^ Spearman rank correlation coefficient.

## Data Availability

Data are available upon reasonable request to the corresponding author.

## Abbreviations

The following abbreviations are used in this manuscript:

AHWTT	Alternate Hand Wall Toss Test
EHC	Eye–Hand Coordination
FNT	Finger–Nose Test
LWT	Loop-Wire Test
PPT	Purdue Pegboard Test
